# Intra-Articular Physiological Saline in Temporomandibular Disorders May Be a Treatment, Not a Placebo: A Hypothesis, Systematic Review, and Meta-Analysis

**DOI:** 10.3390/jcm13216613

**Published:** 2024-11-04

**Authors:** Maciej Chęciński, Kamila Chęcińska, Katarzyna Cholewa-Kowalska, Kalina Romańczyk, Dariusz Chlubek, Maciej Sikora

**Affiliations:** 1 National Medical Institute of the Ministry of Interior and Administration, Wołoska 137 Str., 02-507 Warsaw, Poland; maciej@checinscy.pl (M.C.); sikora-maciej@wp.pl (M.S.); 2 Department of Maxillofacial Surgery, Hospital of the Ministry of Interior, Wojska Polskiego 51, 25-375 Kielce, Poland; 3 Department of Oral Surgery, Preventive Medicine Center, Komorowskiego 12, 30-106 Krakow, Poland; kalina.romanczyk@wp.pl; 4 Department of Glass Technology and Amorphous Coatings, Faculty of Materials Science and Ceramics, AGH University of Krakow, Mickiewicza 30, 30-059 Krakow, Poland; checinska@agh.edu.pl (K.C.); cholewa@agh.edu.pl (K.C.-K.); 5 Faculty of Applied Sciences, WSB Academy, Cieplaka 1C Str., 41-300 Dabrowa Gornicza, Poland; 6 Institute of Applied Sciences, WSB Merito University in Poznan, Sportowa 29 Str., 41-506 Chorzow, Poland; 7 Department of Biochemistry and Medical Chemistry, Pomeranian Medical University, Powstańców Wielkopolskich 72, 70-111 Szczecin, Poland

**Keywords:** temporomandibular joint, temporomandibular joint disorders, intra-articular injections, normal saline, placebo effect

## Abstract

**Background:** Intra-articular injections reduce pain in patients with temporomandibular joint (TMJ) disorders who are unresponsive to conservative treatment. Hyaluronic acid, blood products, and medications provide rapid relief when administered this way, although their mechanisms of action remain unclear. In control groups, which are intended to be untreated, 0.9% NaCl is typically delivered. The hypothesis that “normal saline injections in TMJ cavities produce a therapeutic effect” is proposed, with an exploration of its potential verification, alongside a systematic review and meta-analysis of studies on intra-TMJ 0.9% NaCl. **Methods:** Randomized controlled trials (RCTs) on patients with TMJ internal derangement, arthritis, or degeneration were selected under PRISMA 2020 and assessed with RoB2. **Results:** Seven RCTs with 359 patients were included. Weekly follow-ups revealed a decrease in articular pain by 23.72% (SE: 0.84%; 95% CI: 24.38–21.06%; *p* < 0.01), and monthly follow-ups indicated a decrease of 34.01% (SE: 1.09%; 95% CI: 36.16–31.86%; *p* < 0.01) compared to the baseline values. These findings were grounded in low-risk-of-bias evidence on 267 patients in five RCTs and 222 patients in four RCTs, respectively. **Conclusions:** The hypothesis warrants further testing to determine whether, in addition to the known biological activity of typical injectables, the mechanical action also contributes to pain relief.

## 1. Introduction

Currently, temporomandibular disorders are significant health problems in the population. They are estimated to affect up to 34% of the world’s population [1]. This group is heterogeneous regarding specific diagnoses, including temporomandibular joint (TMJ) internal derangement, inflammation (arthritis), and degeneration [1,2,3,4]. Treatment depends on the cause and severity of the symptoms and includes psychotherapy, physiotherapy, splint therapy, and pharmacotherapy [5,6,7,8,9,10]. If conservative options have been exhausted, an invasive alternative is undertaken: masticatory muscle needling, intra-articular injections, or arthroscopic or open surgeries [11,12,13,14,15]. Intra-articular injections are attractive due to their minimal invasiveness and rapid and long-lasting effectiveness [16,17,18].

In the group of intra-articular injections, we distinguish between joint cavity lavage (arthrocentesis) and intra-articular deposition [19,20,21,22,23]. These two treatment techniques are often combined, with deposition following lavage [18,20]. Typical rinsing agents are physiological solutions, such as Ringer’s lactate and normal saline [24,25]. Hyaluronic acid, autologous blood products, corticosteroids, and other less popular substances are deposited as active agents [12,20,26,27,28]. When clinically evaluating a rinse-and-deposit treatment, typically, the control group receives lavage alone. The effectiveness of intra-articular deposition without prior arthrocentesis is assessed compared to rinsing alone, the deposition of another active substance, or the intra-articular injection of normal saline [29,30,31,32,33,34,35,36,37,38,39]. It is generally accepted that a physiological saline solution is treated as an inactive placebo in the efficacy of injection therapy-controlled clinical trials [29,30,31,32,33,34,35,36,37,38,39]. This assumption, however, is at odds with the noticeably beneficial intervention results in saline placebo groups [29,30,31,32,33,34,35,36,37,38,39].

The mechanism of action of injection therapy for temporomandibular disorders is not fully understood [40,41]. It was established that the TMJ synovial fluid consists mainly of hyaluronic acid, the degradation of which correlates with the intensity of inflammation [7,42,43,44]. It is assumed that arthritis decreases the average molecular weight of hyaluronic acid in the synovial fluid, and shorter hyaluronan chains are less efficient in lubricating joint surfaces, which increases inflammation [42,45,46,47]. Therefore, the supplementation of this substance directly explains the increase in the range of motion in the TMJ and indirectly explains the decrease in articular pain [22,48,49].

After hyaluronic acid, the second best-documented injectable substance is platelet-rich plasma [12,23,42,50]. After its administration, there is no immediate increase in the maximum range of motion in the TMJ [42]. Platelet-rich plasma is considered to have regenerative potential resulting from the content of anti-inflammatory cytokines and growth factors [12,44,51,52,53,54]. Meta-analyses prove an increase in the range of mandibular mobility in the long-term period of 6–12 months following the injection treatment with this blood derivative [55,56]. Pain relief after administering platelet-rich plasma occurs quickly and is statistically significant [23,57]. This aligns with the pain relief effect produced by hyaluronic acid treatment [48].

The mechanisms of the influence of both substances on the range of mandibular mobility are different and result from their biological activity [40,41,48]. However, when injected into the TMJ cavity, hyaluronic acid and platelet-rich plasma provide comparable rapid pain relief [58,59,60]. These discrepancies and similarities are the subject of current research [42,48]. Due to methodological differences, including primarily the popular preceding injection with arthrocentesis and a varying number of administrations, a meta-analytical comparison of the analgesic efficacy of individual agents is challenging [22,32,56,60]. In the context of the above considerations, the authors of this paper noticed that normal saline injected intra-articularly also causes a decrease in pain [29,30,31,32,33,34,35,36,37,38,39]. This can be observed in studies controlled by saline placebo groups when comparing the final pain intensity values with the initial ones [29,30,31,32,33,34,35,36,37,38,39]. To the authors’ knowledge, no one has considered injections of normal saline into the TMJ cavity differently than a placebo control [29,30,31,32,33,34,35,36,37,38,39].

## 2. Main Hypothesis

Based on the observations presented above, we present the following hypothesis:

**Hypothesis** **1.** *Normal saline injections in TMJ cavities produce a therapeutic effect.*

In this paper, we present theoretical considerations supported by previous research, discuss the feasibility of testing the proposed hypothesis, and identify and compare randomized controlled trials in which normal saline was injected into the TMJs.

### 2.1. Theoretical Framework

Normal saline is used, among others, as a body-hydrating substance, a solvent for intravenous drugs, and a fluid for rinsing wounds. The solution is believed to lack biological activity, which may not always be true [61,62,63,64]. A perfect physiological solution should be isotonic to blood and tissue fluid to ensure osmotic balance after administration; regarding 0.9% NaCl, this property is being questioned. The concentration of chloride ions in normal saline is 51 mmol/L higher than that in extracellular fluid [63,64,65]. Some studies highlight that normal saline solutions cause oxidative stress at the tissue level, induce intra-vascular coagulation, and influence endothelial function [61,62]. However, due to its simple composition and low price, it is hard to imagine any medical facility not equipped with sterile saline packages [65].

The assumption of no biological activity has become the probable reason for using saline in control groups of studies assessing the effect of intra-articular injections on the resolution of temporomandibular disorders [29,30,31,32,33,34,35,36,37,38,39]. Technically, injecting saline does not differ from administering other substances into the joint cavity, such as hyaluronic acid, blood products, or drugs [29,30,31,32,33,34,35,36,37,38,39,66]. Typically, researchers also ensure that the volume of substance administered is equal between the active treatment and control groups [29,30,31,32,33,34,35,36,37,38,39].

In a proper control group, the pain should not subside, or its subsidence should result from the placebo effect alone. To the authors’ knowledge, the placebo effect scale in injection therapies has not yet been estimated. However, one research team suspects a nocebo effect following the intra-articular administration of articaine [36,67]. The possible placebo effect is the main threat to the hypothesis and requires validation.

### 2.2. Testability of the Hypothesis

The hypothesis verification may involve calculating the difference between the quantified pain in the groups receiving intra-articular normal saline and non-deposition TMJ punctures. Thus, the analgesic effect of administering 0.9% NaCl into the joint cavity would be confirmed or denied. In both groups, a medical interview, physical examination, qualification, preparation for the intervention, the main elements of the intervention (tissue disruption and placement of the injection needle tip in the joint cavity), visual-analog-scale pain data collection, and clinical checks would be performed.

However, the above verification method is questionable or even unethical. In such a scenario, patients suffering from TMJ pain would be divided into two groups, one of which would intentionally be mutilated and remain untreated. The other would receive an intervention that is generally considered ineffective but may be so in the light of the above hypothesis. Alternatively, there are many injection protocols available that include the administration of biologically active substances, the therapeutic effect of which has been scientifically proven to be stronger than saline. This further undermines the ethics of the proposed study. An animal study with identical assumptions also seems problematic due to measuring pain matters.

An indirect possibility of testing the above hypothesis is a meta-analysis of randomized clinical trials in which groups receiving normal saline were included. However, intra-articular saline typically constitutes a control group for other intra-articular administrations, which does not allow for the independence of the result from the placebo effect. Due to the noninvasiveness and lack of ethical problems, a preliminary literature-based study was undertaken to confirm or refute the validity of designing further experiments or trials.

## 3. Materials and Methods

This systematic review and meta-analysis followed PRISMA 2020 guidelines and PROSPERO registration (CRD42024599439) [68].

### 3.1. Eligibility Criteria, Information Sources, and Search Strategy

The following PICOS eligibility criteria were adopted: (P) patients: TMJ internal derangement, arthritis, or degenerative joint disease, but not in children; (I) intervention: intra-articular injection of normal saline, but without removing the administered fluid (rinsing) and without more extensive treatment (e.g., arthroscopy); (C) comparison: another intra-articular injection, but not Ringer’s lactate; (O) outcomes: articular pain values at baseline and at least one week of follow-up; (S) settings: randomized controlled trials in English, but not preprints [69]. The final searches were carried out on 6 October 2024, in all databases of medical articles covered by Bielefeld Academic Search Engine (BASE), National Library of Medicine PubMed, and Elsevier Scopus engines. We did not limit the time frame. The following query was used: “(temporomandibular OR TMJ) AND (intra-articular OR intraarticular OR intra-cavitary OR intracavitary) AND (injection OR injections) AND (saline OR NaCl) AND (clinical OR patient OR patients) AND (random OR randomized OR randomization)”.

### 3.2. Selection and Data Collection Processes, Data Items, and Study Risk of Bias Assessment

A selection of reports and collection of source data were made by two researchers (M.C. and K.C.), who discussed the discrepancies, and if necessary, the third author (M.S.) had a decisive vote. The initial and follow-up pain values were extracted and transformed to a scale of 0–10. The risk of bias was assessed using the RoB 2 method [70].

### 3.3. Effect Measures, Synthesis Methods, and Certainty Assessment

The ratios of the initial and final pain values were calculated and treated as partial results. Outcomes of individual studies were synthesized and presented along with evidence indicators. MedCalc Software (Version 22.023, MedCalc Software Ltd., Ostend, Belgium) was used to calculate the mean differences, along with standard error (SE), 95% confidence interval (95% CI), and probability (*p*) values. A significance level of α = 0.01 was adopted.

## 4. Results

### 4.1. Study Selection, Characteristics, and Risk of Bias

**Figure 1:**
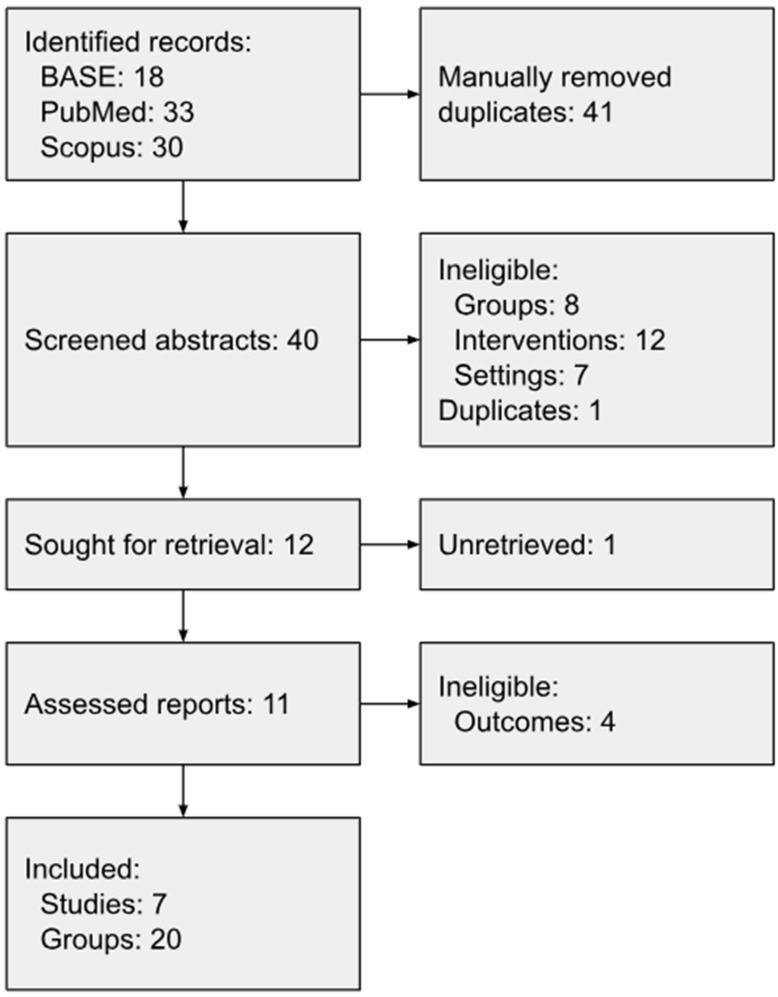
Flow diagram.

Database searches identified 18, 33, and 30 records using BASE, PubMed, and Scopus engines, respectively (Figure 1). We manually removed 41 duplicates before screening and 28 ineligible reports based on titles and abstracts. The unanimous decisions of the assessors excluded 8 reports with inappropriate groups (including animals and patients with incompatible diagnoses), 12 describing another type of intervention (including no deposition of normal saline alone or more invasive treatment such as arthroscopy or arthroplasty), 7 with a design other than a randomized controlled trial (mainly reviews), and 1 previously missed duplicate. Twelve reports were qualified for full-text evaluation. One article from 1991 was not obtained in full text, and the remaining ones are presented in Table 1 [71].

### 4.2. Results of Individual Studies, Synthesis, and Analyses

**Figure 2:**
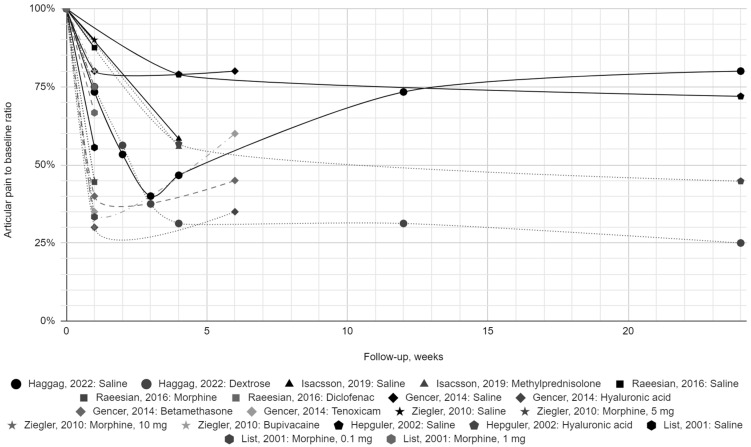
The change in pain severity over time for individual patient groups, presented as a percentage of initial values across the entire follow-up period [29,30,31,32,34,37,38].

**Figure 3:**
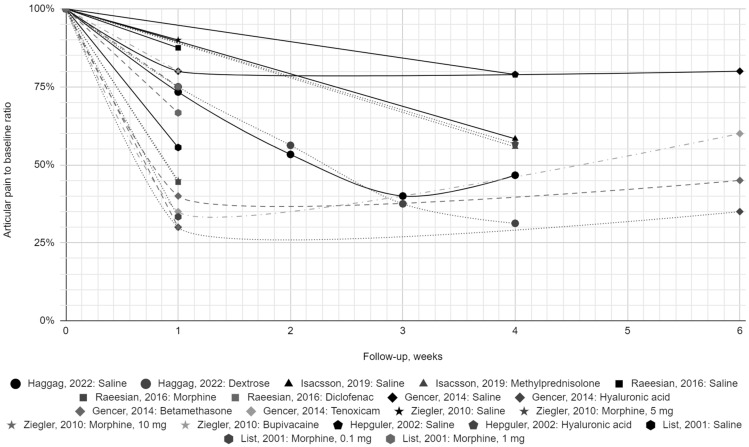
The change in pain severity over time for individual patient groups, presented as a percentage of initial values over the first six weeks of follow-up [29,30,31,32,34,37,38].

Finally, seven reports with a low risk of bias were included in the synthesis. Each provided the results of one group of patients receiving normal saline at a volume of 0.5 to 2 mL, administered from 1 to 4 times (at intervals of up to 7 days). Table 2 presents the values of articular pain intensity before the intervention and during the follow-up period of up to 6 months with respect to the saline and active treatment groups. The change in pain intensity expressed as a percentage of the initial value is presented in Figure 2 and Figure 3.

**Figure 4:**
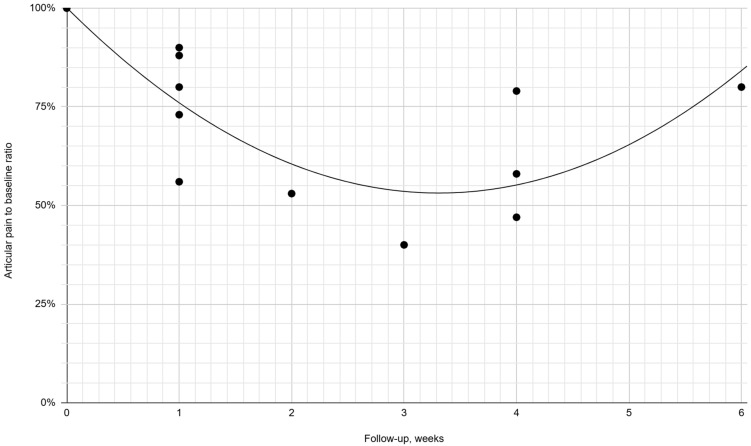
The change in pain severity over the first six weeks for patient groups receiving normal saline as a percentage of initial values with a second-degree polynomial trend (*R*^2^ = 0.58) during this period.

The meta-analytical percentage change from the baseline articular pain severity in the placebo groups is presented over time in Table 3 and Figure 4.

The positive correlations between the number of injections and the volume of normal saline compared to pain after a week of intervention and the negative correlations between the same variables and pain after about a month of intervention emerged (Table 4). The more times the greater volume of normal saline was injected, the higher the pain remained after a week, but the stronger it decreased after about a month of administration. Moreover, the stronger the pain before the intervention, the weaker the therapeutic effect achieved after a week, but this relationship decreased significantly after about a month of follow-up.

### 4.3. Certainty of Evidence

For the most frequently represented observation periods, i.e., 1 week and approximately 1 month, the mean difference in pain intensity from baseline was calculated along with certainty indices (Table 5). The one-week follow-up mean difference was −23.7% in the groups receiving normal saline. In all meta-analyzed materials, the change in pain ranged from −10.0% to −70.0%, with a mean of −41.2%. After about one month, pain in the normal saline groups changed from the baseline by −34.0%, and in all samples, it changed by −45.2% from −20.0% to −68.8%.

## 5. Discussion

### 5.1. General Interpretation of the Results

Considering varying administration protocols, observation lengths, and follow-up visit frequencies and the use of different therapeutic agents in the active treatment groups, comparing the results of individual reports is challenging.

The identified studies differed in normal saline administration protocols. A single injection consisted of 0.5 to 2 mL of fluid, which could have increased hydrostatic pressure in the joint. The TMJ cavity assessed radiologically has an average volume of 1.8 mL [72]. According to magnetic resonance imaging, superior and inferior TMJ compartments contain 1.2 mL and 0.9 mL of synovial fluid, respectively [73]. Both compartments may undergo intra-articular injection [74,75]. We suppose that the injection of any substance stretches the joint capsule and exerts pressure on cartilage cells.

Another source of heterogeneity was the number of injections making up the entire intervention and the length of the intervals between them. In the included studies, investigator teams proposed 1 to 4 administrations [29,30,31,32,34,37,38]. Another one, rejected due to the insufficient reporting of results, documented five injections [33]. Intervals did not exceed 7 days [30,33,37]. The identified protocols did not differ from those commonly used [8,22,23]. The more difficult to scientifically interpret but common approach is to repeat injections until a therapeutic effect is achieved [30].

The length of post-intervention observation ranged from 1 week to 6 months [29,30,31,32,33,34,37,38,39]. Following the established eligibility criteria, studies with shorter follow-ups were excluded [35,36]. One of the rejected reports limited observation to as low as 5 min, which was methodologically justified but prevented the observation of the effect sought in this meta-analysis [36]. No dominant follow-up schedule was perceived. However, measurement points following the passage of generally accepted time units, such as 1 week and 1 month, are commonly provided [29,30,31,32,34,37,38]. Due to their larger representation, they were retained for the summary of findings [29,30,31,32,34,37,38].

The baseline average pain intensity varied in each normal saline group; therefore, we presented the initial pain value as 100% in graphical synthesis. It revealed a 10.0–44.4% relief in the first week after intra-articular saline injection [30,31,32,34,38]. Despite the numerical variability, pain consistently improved within each group, with no outlines of no change or worsening [30,31,32,34,38]. The meta-analytical mean pain score reached its lowest value of 40.0–61.3% 2–4 weeks post-intervention, showing an improvement of 38.7–60.0% [29,30,37]. The mean meta-analytic improvement over this period in the active treatment groups at 2–4 weeks of follow-up was 43.8–62.5% [29,30,37]. This is consistent with the results for the 0.9% NaCl groups, although based on only three studies [29,30,37]. These included hypertonic dextrose, methylprednisolone, and hyaluronic acid administrations [29,30,37]. During further follow-up, the improvement in actively treated groups was maintained and even increased to 68.8% [30,32,37]. The situation differed in patients receiving saline, as pain values returned to 73.3–80.0%, i.e., the improvement was limited to 20.0–26.7% [30,32,37]. However, partial pain recurrence in saline controls did not exceed 80.0% of the baseline value in any data series at any subsequent time point. The observed weak but stable effect of intra-articular physiological saline prompts further research.

Rapid pain relief, possible partial relapse, and persistent therapeutic effects over time are characteristics of injection therapy with conventional agents such as hyaluronic acid and platelet-rich plasma [22,23,32,37]. In other words, the visual representation of pain change for individual groups receiving normal saline is consistent with the graphs for groups treated with generally recognized agents [30,31,32,34,38]. All included studies were controlled, randomized, and blinded [29,30,31,32,34,37,38]. Despite protocol differences, they produced results similar to each other and the active treatment groups [29,30,31,32,34,37,38]. This similarity consisted of the aforementioned sudden decrease in pain in the first period and further maintenance at a level lower than before treatment [29,30,31,32,34,37,38]. Individual active substances show their therapeutic effects, giving some of them an advantage over normal saline [30,32,37]. In the context of the above results and considerations, this may be an addition to the mechanism of action in common with saline.

The effectiveness of TMJ cavity rinsing (arthrocentesis) is based on inflammatory mediator removal and adhesion break mechanisms [21,25,76,77]. The first involves flushing synovial fluid from the joint and enhancing new fluid creation or enabling injection [21,76,77]. Intra-TMJ administration without rinsing may improve the composition of synovial fluid as part of the biological activity of the injected substance [42,48]. It can be assumed that adding even a biologically neutral solution to the joint cavity reduces the concentration of inflammatory mediators, hypothetically resolving arthritis. The second mechanism seems common to arthrocentesis and intra-articular injections of any substance; The administration of fluid under pressure into the joint cavity mechanically affects adhesions [21,76,77]. In the current state of knowledge, these mechanisms constitute auxiliary hypotheses of the action of intra-TMJ normal saline.

In summary, it was observed that regardless of whether saline or an active compound was administered, the severity of the pain remained decreased. It remains unresolved if this represents a placebo effect consistently observed across studies or suggests a previously unexplored mechanism of action.

### 5.2. Limitations of the Evidence and Review Processes

Despite extensive searches of medical literature databases, the number of randomized controlled trials that included the administration of normal saline in controls was scarce [29,30,31,32,33,34,35,36,37,38,39]. About half of the identified reports were published at least 10 years ago [32,34,37,38]. This is probably due to the popularization of controlling studies with joint lavage or alternative active substance administration [18]. The injection of normal saline is interpreted by the research teams that perform it as no treatment [29,30,31,32,33,34,35,36,37,38,39]. It hinders recruiting patients who do not want to risk qualification for the placebo group.

The included studies were heterogeneous in many of the aspects already discussed. Different observation protocols resulted in significant gaps in the tabular synthesis. This resulted in assuming the inter-value course of the pain intensity change curves in the graphical outcomes’ representation. Most reports did not present standard deviations from mean values of joint pain; therefore, these values had to be completely omitted. This significantly limited the analysis and prevented visualization using a forest plot.

Due to the limitations of our research team, only English-language reports were included in the review.

### 5.3. Implications for Future Research

A similar systematic review to this one was conducted for the injection treatment of back pain [78]. In randomized controlled trials, pain relief was achieved with normal saline administered to facet joints [78]. Meta-analyses showed preliminary evidence of comparable therapeutic effects to substances considered active treatments [78].

In the context of all the above considerations, administering any fluid, including 0.9% NaCl, into the TMJ cavity may impact articular tissues. Normal saline can influence tissues in many ways: through mechanical pressure as a rinsing agent, and, according to some researchers, it can even alter the osmotic balance. This issue seems amenable to further investigation in studies on cell lines and animals.

In addition, an intra-articular fluid injection may shape the pain-in-time curve. Presumably, it can be further modified by adding an active substance to the injectable composition. It is advisable to undertake frequent, preferably weekly, assessments of articular pain intensity in all studies on injection therapies for temporomandibular disorders. This will facilitate the future meta-analytical search for a common part between pain curves. The hypothetical pattern may correspond to injecting any liquid into the joint cavity filled with natural synovial fluid.

## 6. Conclusions

The hypothesis on the therapeutic effect of intra-articular injections of normal saline in temporomandibular disorders is challenging to verify. In light of the conducted meta-analysis and the considerations undertaken, it is presumed that the observed decrease in pain intensity after such an intervention may result from mechanical action, the placebo effect, or a slight biological impact. The convergence of pain intensity change curves over time following the administration of both active substances and normal saline suggests a common effect of these procedures.

## Figures and Tables

**Table 1:** Reports assessed in full text.

The First Author of the Report, Publication Year	Digital Object Identifier	Number of Patients/Joints in the Entire Sample	Number of Injections/ Interval Duration (Days)	Saline Volume per Injection (mL)	Substances in Active Treatment Groups	Risk of Bias in Subsequent Domains—Total Risk of Bias	Decision
Haggag, 2022 [30]	10.1016/j.jcms.2022.02.009	30/60	0–4/7	1.0	25% dextrose	0/0/0/0/0—Low	Included
Isacsson, 2019 [29]	10.1111/joor.12718	54/54	1/N/A	1.0	Methylprednisolone	0/0/0/0/0—Low	Included
Raeesian, 2016 [31]	10.22037/rrr.v1i3.10664	36/36	2/2	2.0	Morphine, diclofenac	0/0/0/0/0—Low	Included
Gencer, 2014 [32]	10.1016/j.jcms.2014.01.041	100/N/S	1/N/A	0.5	Hyaluronic acid, Betamethasone, Tenoxicam	0/0/0/0/0—Low	Included
Tang, 2010 [33]	10.1016/j.tripleo.2009.11.007	40/N/S	5/7	1.0	Hyaluronic acid	0/0/0/0/0—Low	Excluded due to lack of post-treatment pain values
Ziegler, 2010 [34]	10.1016/j.joms.2009.04.049	48/N/S	3/2	2.0	Morphine	0/0/0/0/0—Low	Included
Ayesh, 2008 [35]	10.1016/j.pain.2007.09.004	18/N/S	1/N/A	0.2	Ketamine	0/0/0/0/0—Low	Excluded due to follow-up limited to 1 day
Tjakkes, 2007 [36]	10.1097/ajp.0b013e31802f0950	19/19	1/N/A	0.5	Articaine	0/0/0/0/0—Low	Excluded due to follow-up limited to 5 min
Hepguler, 2002 [37]	10.1046/j.1365-2842.2002.00807.x	38/38	2/7	0.5	Hyaluronic acid	0/0/0/0/0—Low	Included
List, 2001 [38]	10.1016/s0304-3959(01)00361-x	53/53	1/N/A	1.0	Morphine	0/0/0/0/0—Low	Included
Bertolami, 1993 [39]	10.1016/s0278-2391(10)80163-6	121/122	1/N/A	N/S	Hyaluronic acid	0/0/0/0/1—Some concerns	Excluded due to no pain value reporting

0—low risk of bias; 1—some concerns; 2—high risk of bias; N/A—not applicable; N/S—not specified.

**Table 2:** Baseline and follow-up pain values in groups intra-articularly receiving normal saline or active treatment.

The First Author of the Report, Publication Year	Administered Substance	Number of Patients/Joints	Baseline	1 Week	2 Weeks	3 Weeks	4 Weeks	6 Weeks	12 Weeks	24 Weeks
Haggag, 2022 [30]	Saline	15/30	7.5	5.5	4.0	3.0	3.5		5.5	6.0
Haggag, 2022 [30]	Dextrose	15/30	8.0	6.0	4.5	3.0	2.5		2.5	2.0
Isacsson, 2019 [29]	Saline	27/27	6.0				3.5			
Isacsson, 2019 [29]	Methylprednisolone	27/27	6.1				3.4			
Raeesian, 2016 [31]	Saline	12/12	8.0	7.0						
Raeesian, 2016 [31]	Morphine	12/12	9.0	4.0						
Raeesian, 2016 [31]	Diclofenac	12/12	8.0	6.0						
Gencer, 2014 [32]	Saline	25/N/S	10.0	8.0				8.0		
Gencer, 2014 [32]	Hyaluronic acid	25/N/S	10.0	3.0				3.5		
Gencer, 2014 [32]	Betamethasone	25/N/S	10.0	4.0				4.5		
Gencer, 2014 [32]	Tenoxicam	25/N/S	10.0	3.5				6.0		
Ziegler, 2010 [34]	Saline	12/N/S	10.0	9.0						
Ziegler, 2010 [34]	Morphine, 5 mg	12/N/S	10.0	4.5						
Ziegler, 2010 [34]	Morphine, 10 mg	12/N/S	10.0	3.0						
Ziegler, 2010 [34]	Bupivacaine	12/N/S	10.0	8.0						
Hepguler, 2002 [37]	Saline	19/19	5.7				4.5			4.1
Hepguler, 2002 [37]	Hyaluronic acid	19/19	6.7				3.8			3.0
List, 2001 [38]	Saline	18/18	4.5	2.5						
List, 2001 [38]	Morphine, 0.1 mg	17/17	4.5	1.5						
List, 2001 [38]	Morphine, 1 mg	18/18	4.5	3.0						

N/S—not specified.

**Table 3:** The change in pain severity over time for patient groups receiving normal saline.

	Baseline	1 Week	2 Weeks	3 Weeks	4 Weeks	6 Weeks	12 Weeks	24 Weeks
Mean articular pain to baseline ratio	100.0%	77.4%	53.0%	40.0%	61.3%	80.0%	73.3%	76%
Standard deviation	N/A	13.7%	N/A	N/A	16.3%	N/A	N/A	5.7%

N/A—not applicable.

**Table 4:** Pearson *r* correlation test result’s matrix.

	Number of Saline Injections	Saline Volume per Injection (mL)	Baseline Pain	Pain After 1 Week	Pain After Approximately 1 Month
Number of saline injections	1.00	0.63	0.44	0.69	−0.23
Saline volume per injection (mL)	0.63	1.00	0.35	0.58	−0.96
Baseline pain	0.44	0.35	1.00	0.87	0.24
Pain after 1 week	0.69	0.58	0.87	1.00	1.00
Pain after approximately 1 month	−0.23	−0.96	0.24	1.00	1.00

**Table 5:** The summary of findings, including mean differences between percentage improvements in the normal saline groups and baseline pain intensity values.

Follow-Up Duration	Number of Studies	Maximal Risk of Bias in Studies	Total Number of Patients	Mean Difference	Standard Error	Lower 95% Confidence Interval	Upper 95% Confidence Interval	Probability Values
1 week	5	Low	267	−23.72%	0.84%	−24.38%	−21.06%	*p* < 0.01
4–6 weeks	4	Low	222	−34.01%	1.09%	−36.16%	−31.86%	*p* < 0.01

## Data Availability

The original contributions presented in the study are included in the article. Further inquiries can be directed to the corresponding author.
